# Renal functional outcomes after surgery for renal cortical tumors

**DOI:** 10.15586/jkcvhl.2015.26

**Published:** 2015-04-04

**Authors:** Danny Lascano, Julia B. Finkelstein, G. Joel DeCastro, James M. McKiernan

**Affiliations:** Herbert Irving Cancer Center, Columbia University College of Physicians and Surgeons, Department of Urology, New York, NY, USA

## Abstract

Historically, radical nephrectomy represented the gold standard for the treatment of small (≤ 4cm) as well as larger renal masses. Recently, for small renal masses, the risk of ensuing chronic kidney disease and end stage renal disease has largely favored nephron-sparing surgical techniques, mainly partial nephrectomy. In this review, we surveyed the literature on renal functional outcomes after partial nephrectomy for renal tumors. The largest randomized control trial comparing radical and partial nephrectomy failed to show a survival benefit for partial nephrectomy. With regards to overall survival, surgically induced chronic kidney disease (GFR < 60 ml/min/ 1.73m^2^) caused by nephrectomy might not be as deleterious as medically induced chronic kidney disease. In evaluating patients who underwent donor nephrectomy, transplant literature further validates that surgically induced reductions in GFR may not affect patient survival, unlike medically induced GFR declines. Yet, because patients who present with a renal mass tend to be elderly with multiple comorbidities, many develop a mixed picture of medically, and surgically-induced renal disease after extirpative renal surgery. In this population, we believe that nephron sparing surgery optimizes oncological control while protecting renal function.

## Introduction

Renal lesions can be classified as malignant, benign, or inflammatory. Inflammatory renal lesions may mimic malignant renal lesions on imaging and include infection, inflammation, or trauma induced lesions (1). Of the non-inflammatory cases, benign masses compose approximately 13% of newly diagnosed lesions such as oncocytomas and angiomyolipomas; the rest renal cell carcinoma (2). Renal cell carcinoma (RCC) accounts for 3.8% of all cases of adult malignant neoplasms. It typically presents in the sixth and seventh decades of life. RCC of clear cell histology is the most common, followed by papillary and chromophobe subtypes (2). Overall, the incidence of RCC has increased in the last three decades with an estimated 63,920 cases and 13,860 deaths (3). The advent of improved imaging techniques such as computer tomography (CT) and magnetic resonance imaging (MRI) has partially driven this rising incidence, as clinicians can now detect pre-symptomatic renal tumors incidentally (3, 4). Accordingly, small renal masses (SRM) that are less than or equal to 4 cm are being detected more frequently. In the prior decade, the average renal tumor size decreased from 6.7 cm to 5.9 cm (5).

In part, imaging can assist in differentiating renal masses of unknown malignant potential. For instance, benign lesions like angiomyolipomas can be identified by the presence of macroscopic fat. CT or MRI scan with intravenous contrast administration can help distinguish those renal masses that need further evaluation. For a renal mass to be considered malignant, it should enhance with administration of contrast. However, 10–20% of small, solid CT-enhancing renal masses are found to be benign after surgical removal (6). In particular, differentiating a benign renal cyst and a cystic RCC by imaging is difficult.

In terms of the size distribution of RCC, 35% of tumors are < 4 cm, 33% are between 4 and 7 cm, and 32% are > 7 cm (5). Larger masses are increasingly correlated with malignancy and worse outcomes (7). The size of the renal mass, tumor risk profile, and clinical symptoms are all significant prognostic factors. However, pathologic stage is the most important prognostic factor. Patient-related factors like comorbidities and frailty are also influential in determining appropriate management.

In the management of a renal mass the most important predictors of post-operative GFR besides pre-operative GFR are both residual functioning parenchyma and ischemia time (8). Chronic kidney disease (CKD) in general is defined as an estimated glomerular filtration rate (GFR) of less than 60 mL/min/1.73m^2^ for over 90 days (9). The different stages of CKD are categorized as shown in **Table 1**. End stage renal disease (ESRD) is defined as GFR less than 15 mL/min/1.73m^2^ and requires renal replacement therapy such as hemodialysis.

**Table 1.** Definitions of CKD stages based on GFR

Chronic Kidney Disease Stage	Estimated GFR (ml/min/ 1.73m^2^)
I	≥ 90
II	60 – 89
III	30 – 59
IV	15 – 29

 As we progress beyond the Halstedian era of radical extirpative approaches in oncologic surgery and move into the era of minimally invasive surgery, a series of questions arise in the management of renal masses. One specific question that we will address is whether sparing nephrons impacts mortality.

## Management Approaches

As stated above, localized SRMs have increased in incidence and now are a fairly common clinical situation. Historically, radical nephrectomy represented the gold standard for the treatment of all renal masses. The first documented radical nephrectomy was completed for the treatment of renal cell carcinoma in 1963 (10). It still represents the standard of care in non-localized cases and for renal masses of unknown malignant potential in 30% of cases (11). However, practices have changed dramatically in the last two decades. It has been recognized that SRMs have broad heterogeneity in tumor biology and several management strategies are now offered, including radical nephrectomy (RN), partial nephrectomy (PN), thermal ablation (TA) as well as active surveillance (AS). Moreover, for treating SRMs, the risk of ensuing CKD and ESRD requiring renal replacement therapy has largely favored nephron-sparing surgery.

PN involves complete but localized resection of the tumor, while maintaining the most amount of normal parenchyma possible. For the surgical management of SRMs of ≤4 cm, PN has become standard of care. Some even suggest its application be expanded to masses up to 7 cm in size, given their 20–30% likelihood of benign pathology (12). With regards to approach, both laparoscopic and robotic PNs have been shown to have good outcomes with short recovery time, acceptable ischemia time, and less morbidity than open PN (13, 14). Robotic technology is generally preferred for PN, given the technical limitations of laparoscopic surgery, and the literature does support its use for moderate to complex renal masses given the decreased conversion rate to RN for robotic PN in comparison to laparoscopic PN (15).

Thermal ablative treatments such as renal cryoablation (CA) and radiofrequency ablation (RFA) have materialized as alternative nephron-sparing therapies for patients with localized SRMs. Both techniques can be initiated percutaneously or via laparoscopic exposure. Some report reduced morbidity with this treatment but the long-term oncological control has not been well established, with a greater incidence of local recurrence reported for these techniques than for surgical approaches. RFA is reported to have a likelihood of tumor recurrence of 12.9% and risk of metastasis of 2.5%, even within a well-selected population (16). Meanwhile, a meta-analysis by Kunkle and Uzzo looking at CA showed a likelihood of tumor recurrence of 5.2% and risk of metastasis of 1% (16). These TA recurrences may be salvageable with repeat ablation, although some need traditional surgery. In the latter case, radical or partial nephrectomy may be impossible to perform secondary to the widespread fibrotic reaction caused by the TA (17).

However, the same population that may benefit from ablative treatment of SRMs, may benefit from inclusion into an active surveillance with delayed intervention protocol (18). Bosniak et al showed that renal tumors grow at slow and variable rates of up to 1.1 cm per year with a median growth rate of 0.36 cm per year (19). A more recent study by Crispen and colleagues that followed patients with a localized, enhancing renal mass revealed that absolute growth rate following detection of the tumor was 0.039 cm/year (20). In another study observing 209 patients with SRMs and limited life expectancy for a mean of 28 months, local progression occurred in 12% and 2 patients (1.1%) developed metastases (21). Besides the slow growth rate and limited progression of most SRMs, the risk of competing causes of death and of intervention may also favor AS in this population. A study by Hollingsworth et al. evaluating patients’ survival 5 years after surgical treatment of RCC showed that one third of the elderly may die from their comorbidities (22). Therefore, elderly patients or patients of poor surgical risk with a small, solid, well-defined renal lesion may be managed with active surveillance, involving serial renal imaging biannually or annually, and delayed intervention when necessary.

## Renal function after TA techniques and on AS

Some literature endorses superior renal function with TA over conventional surgery. A study by Woldu et al. comparing renal parenchymal loss between CA, RFA, and PN showed that TA was associated with less renal parenchymal loss (23). In another retrospective comparison of patients with a suspicious renal mass of less than 5 cm, Lucas et al revealed that RFA has a freedom of CKD of 95.2% in comparison to PN at 70.7% and RN at 39.9% (24). In a European study evaluating cryoablation, renal function was relatively well conserved, as prior to treatment GFR was 66 mL/min and it was 60 mL/min post-CA (25). In addition, those with existing CKD experienced no change in GFR.

Limited data exists on renal function while on active surveillance. In a recent analysis from the Delayed Intervention and Surveillance for Small Renal Masses Registry (DISSRM), in a group of 64 patients on AS with a renal mass of < 4 cm and a median baseline GFR of 70.3, 64% of patients experienced a decline in GFR at a yearly rate of 1.82 mL/min/1.73m^2^. This GFR decline is higher than would be expected from aging alone. Furthermore, 24% of patients in the study experienced upstaging in their CKD classification (26). However, given the multiple comorbidities and advanced age of many patients who present with a SRM, AS remains an attractive alternative that warrants further investigation.

## Renal Function after Extirpative Surgery

Most of the literature has focused on renal function after radical and partial nephrectomy. A main concern with performing RN is reduction of GFR and ultimately requiring dialysis. In a retrospective study of 290 patients with SRMs < 4 cm, McKiernan, et al. showed that 5-year freedom from chronic renal insufficiency, which was defined as a creatinine of > 2 mg/mL, was 100% in the PN group and 84.6%% in the RN group (27). In another retrospective study, Huang and colleagues revealed that 65% of RN patients, in comparison to 20% of the PN patients, had grade III CKD (GFR < 60 ml/min/1.73m^2^) at 3-year follow-up (28). Severe CKD was also more likely after RN than PN, with an incidence of 36% versus 5% respectively. In other studies, when the tumor mass and pre-operative GFR was taken into account the loss of kidney function remained higher in RN than PN (29, 30).

Furthermore, a retrospective study by Kaushik and colleagues evaluated patients undergoing RN or PN for a benign renal mass, which eliminates the confounder of malignancy in the survival equation. They demonstrated that overall survival at ten years was 69% for RN and 80% for PN, with a decreased risk of CKD in the PN group in comparison to RN group (31). This alludes to a possible superiority of NSS over RN with regards to renal function. Finally, one of the largest and most recent studies evaluating 2068 patients with a 5-year follow up period showed that renal function after RN led to new onset CKD stage III in 36.1% of patients and new onset CKD stage IV in 3.4% of patients (32).

Ischemia is the major concern with PN, as this may induce tissue necrosis and irreversible damage to the functioning renal parenchyma. This is especially pronounced in cases where ischemia is more than 40 minutes, although even in shorter intervals there is some evidence of parenchyma atrophy (33). However, whether reducing ischemia time leads to a reduction in nephron damage as measured by GFR function is unclear. A recent meta-analysis by Liu et al. revealed that there was a higher odds of GFR decline in patients who undergo on-clamp partial nephrectomy in comparison to off-clamp partial nephrectomy without ischemia (34). Yet, no study thus far has prospectively looked at the post-operative renal function of off-clamp versus on-clamp with ischemia.

Nevertheless, the largest randomized control trial comparing RN and PN failed to show a survival benefit of NSS. In the EORTC 30904 trial, Van Poppel and colleagues demonstrated that 85.7% of patients undergoing RN experience a reduction in their GFR to below 60 ml/min/ 1.73m^2^ in comparison to only 64.7% of the group undergoing PN (35, 36). Despite this diminished impact on renal function, the PN group did not experience improved overall mortality outcomes. In other words, the higher incidence of de-novo CKD post-surgery in the RN cohort did not portend greater overall mortality. Since the European population has a lower level of comorbidities in comparison to an American population, this study was more accurately evaluating the impact of surgical CKD (CKD-S). Perhaps, with regards to overall survival, CKD-S caused by nephrectomy might not be as deleterious as medical CKD (CKD-M).

## Defining surgical versus medical chronic kidney disease

Traditionally, literature on CKD has focused on medical CKD-M, which affects over 19 million Americans (37). This type of CKD stems from microscopic damage at the level of nephrons, either from hypertension, diabetes, or other medical causes. CKD-M increases the risk of death, mainly from adverse cardiovascular events (38). In addition, CKD has been associated with coagulopathies, anemia, left ventricular hypertrophy, arterial calcification, and other pathophysiology (39–43). Most importantly, CKD places patients at risk for ESRD and its accompanying high rate of mortality, morbidity, and cost to the healthcare system (44).

Only the urological and transplant literature distinguish surgical CKD-S from medical causes of renal dysfunction. CKD-S as defined by Lane et al is when patients develop chronic renal insufficiency after nephrectomy without an underlying medical cause for their renal disease (45). Because patients who present with a renal mass tend to be elderly with multiple comorbidities, many develop a mixed picture of CKD-M and CKD-S after extirpative renal surgery (46). This was confirmed by the landmark study from Memorial Sloan Kettering Cancer Center discussed above (45). Twenty-six percent of 662 patients with a small solitary tumor had preexisting grade III CKD. Furthermore, in a retrospective study of 4180 patients undergoing nephrectomy of any type, Lane and colleagues showed that the annual decline in GFR for patients with existing CKD-M who develop CKD-M/S was 4.7% after surgery (45). On the other hand, for those without pre-existing CKD who developed CKD-S, the decline was only 0.7% in GFR. Post-operative GFR was not a significant predictor of survival after 6.6-year median follow-up for patients with CKD-S but did predict survival in those with CKD-M/S with worse survival for those with lower post-operative GFR. This data was supported by another study from the same group in which CKD-M/S and CKD-S groups were compared to those with CKD-M who did not undergo surgery. Demirjian and colleagues showed that the CKD-S group had better overall survival and less of a decline in renal function (47). This validates that CKD-S is a separate entity from CKD-M and mixed CKD-M/S. It follows that urological experience with CKD-S may parallel that of the donor nephrectomy population analyzed in the transplant literature.

## The pathophysiology of surgical CKD and review of the transplant literature

The hypothesized mechanism for renal injury after renal transplant in the remaining donor’s kidney is renal hyperfiltration possibly followed or preceded by renal hypertrophy. Animal models as well as research on human renal tissue show that after nephron loss there is a concomitant increase in the GFR of the remaining kidney (48, 49). It is hypothesized that given the decline in the number of nephrons, the remaining kidney tissue hypertrophies leading to increased renal volume due to the increase of renal plasma flow and increased intraglomerular pressure (50). Eventually the nephrons become unable to compensate with the increased load leading to nephron exhaustion (51). Brenner and colleagues propose that this increased hyperfiltration and the decrease in nephron number may explain why some patients develop renal injury, hypertension, proteinuria and other kidney related diseases (52).

However, since not all patients develop this adverse pathology or a significant GFR decline after surgery, there must be a further explanation. There may be a threshold below which a kidney can tolerate further strain— that is a nephron reserve defined by the nephron surface area and mass (53). Once this reserve is overwhelmed, perhaps damage becomes unmanageable with ensuing kidney function decline. The evidence for this theory largely stems from animal studies, retrospective papers, and one prospective study. Brenner et al in a rat model showed that after thermal renal ablation of a renal mass the remaining nephrons experience hypertrophy on pathology (54). From this experiment, it was hypothesized that the increase in GFR with concomitant low nephron reserve leads to increased intraglomerular hypertension and eventually albuminuria and kidney function decline in humans also (55). This increase in GFR measured by higher than normal rates has been shown to occur in patients with unilateral renal agenesis, congenitally reduced nephron numbers, and acquired reduction in renal mass (56–58). Elsherbiny and colleagues suggest that increased renal plasma flow may induce renal damage that eventually leads to glomerulosclerosis, GFR decline, and hypertension (59). Their study looked at nephron size using biopsies obtained from donor kidneys during transplantation and showed that indeed some of these predicted structural characteristics of hyperfiltration are seen in humans pre-operatively in patients with high GFR at time of their biopsy. Moreover, larger glomerular volume, increased mean profile tubular area, and lower glomerular density were all associated with risk factors for CKD (59).

To be a kidney donor, stringent criteria must be met including having a high baseline GFR and minimal to no comorbidities. The transplant literature has analyzed survival in these patients who have donated their kidney. This population may most accurately reflect CKD-S. In a large cross-sectional study among older kidney donors, Fehrman-Ekholm et al showed that 10% developed proteinuria and half of the male donors developed hypertension (60). Both of these results are higher than expected in the general non-donor population. Overall, 72% of the group had a decline in their average estimated GFR based on their age. Out of 402 donors who lived to follow up, only 5 patients developed a GFR of less than 30 and 1 patient ultimately required dialysis. Ibrahim and colleagues evaluated the incidence of ESRD after unilateral donor nephrectomy and found that 14.5% of their cohort developed CKD with a GFR of less than 60 ml/min/ 1.73m^2^ at most recent follow up. They also noted a higher than expected incidence of hypertension and albuminuria, but overall survival did not differ between kidney donors and matched non-donors (61). This further suggests that surgically induced reductions in GFR may not affect patient survival, unlike medically induced declines. In addition, this data may elucidate why the EORTC 30904 failed to show a survival benefit for PN despite the increased CKD in the RN cohort.

## Future directions

Other than renal biopsy, there is currently no mechanism that predicts what the pathology of a renal mass will be. Both advances in imaging and development of biomarkers that can be correlated with histology are necessary to help differentiate renal masses. This would prevent the surgical removal of a substantial number of tumors that are actually benign or of low malignant potential. It could also guide in selecting the appropriate management strategy based on tumor risk profile along with patient characteristics. Improving our assessment of kidney function beyond GFR would also assist in more aptly risk stratifying patients.

Furthermore, research should be dedicated to resolving the question of whether RN is superior to PN in terms of overall survival in a more heterogeneous population. Ideally, another randomized controlled trial should be completed. Along these lines, further evaluation of the alternative nephron-sparing techniques and their oncological as well as renal functional impact is necessary. Studies with longer-term follow up are needed for thermal ablation and active surveillance.

Finally, a better grasp of the pathophysiology of surgically induced chronic kidney disease is warranted. Further understanding of the mixed state of medically and surgically induced CKD in the aging population is also necessary. While surgically induced CKD seems to be a separate entity with different mortality rates, the literature currently makes little or no distinction.

## Conclusions

In patients with small renal masses, a solitary kidney, multiple comorbidities, or those with multiple tumors, nephron-sparing surgery, mainly partial nephrectomy, is considered standard of care. Thermal ablative treatments have materialized as alternative nephron-sparing therapies for patients with localized small renal masses. These therapies have been associated with higher recurrence rates and have unknown long-term oncological outcomes. Therefore, of the nephron-sparing treatments, we would argue that partial nephrectomy optimizes oncological control while protecting renal function.

Nonetheless, a large randomized controlled trial comparing radical and partial nephrectomy failed to show a survival benefit of nephron sparing surgery. This finding as well as data from the kidney donor population indicates that surgically induced renal dysfunction may not warrant as much concern or vigilance as medically induced renal disease. Further investigation and randomized trials are warranted to help elucidate the benefits of PN in comparison to RN as well to explore the pathophysiology and impact of medically versus surgically induced chronic kidney disease.
